# Strain‐ and sex‐dependent pulmonary toxicity of waterpipe smoke in mouse

**DOI:** 10.14814/phy2.13579

**Published:** 2018-02-07

**Authors:** Naushad Ahmad Khan, Isaac Kirubakaran Sundar, Irfan Rahman

**Affiliations:** ^1^ Department of Environmental Medicine University of Rochester Medical Center Rochester New York

**Keywords:** Inflammation, lipid peroxidation, oxidative stress, glutathione, waterpipe (hookah)

## Abstract

Waterpipe smoking is emerging as a form of tobacco smoking, but its lung health/risks is not known. It has been shown that different mouse strains show differences in susceptibility to tobacco smoke. However, the effect of waterpipe smoke (WPS) exposure and strain differences in susceptibility to oxidative and inflammatory responses is not known. Here, we showed acute WPS exposure induced oxidative stress and inflammatory response in C57BL/6J and BALB/cJ mouse strains. WPS exposure induced inflammatory cell influx (neutrophils and T‐lymphocytes) in bronchoalveolar lavage fluid (BAL fluid), which varied among mouse strains. Proinflammatory cytokines release differed among both the strains, but was significantly increased in C57BL/6J mice. Myeloperoxidase levels in BAL fluid were increased significantly in both the strains. Total reduced glutathione (GSH) level was decreased, whereas the level of oxidized or glutathione disulfide (GSSG) increased in lungs of both the strains. Similarly, the level of lipid peroxidation markers, 15‐isoprostane (plasma), malondialdehyde and 4‐hydroxy‐2‐nonenal (lung homogenates) were increased by WPS. Our data suggest that, oxidative stress and inflammatory responses are influenced by strain characteristics during acute WPS exposure. Overall, C57BL/6J mice showed more susceptibility to oxidative stress and inflammatory responses compared to BALB/cJ mice. Acute WPS mediated pulmonary toxicity is differentially regulated in different mouse strains.

## Introduction

**Figure 1:**
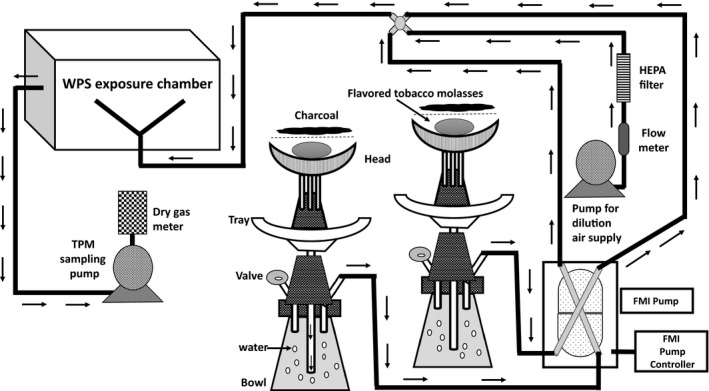
Schematic of laboratory waterpipe/hookah exposure setup for in vivo mouse exposure model. The waterpipe/hookah smoking system is setup for mouse exposure, which contains two waterpipe smoking assembly connected to the FMI pump that controls the timed flow of WPS along with dilution air into the exposure chamber using a computer controlled program. The pump is setup to a defined puff topography (e.g., ~171 puffs/h, each puff is about 3−4 sec. long with 17 sec. puff interval) as reported previously (Hakim et al. 2011). The flow rate of the pump which draws WPS was setup to 1.0 L/min with a constant flow of dilution air (filtered air) into the chamber (at the rate of 0.6 L/min). The exposure chambers were made of plexiglass, containing wired cages with cubical for individual mice. The arrows in the schematic indicates directionality of WPS and dilution air flow into the exposure chamber. WPS, Waterpipe Smoke

Waterpipe smoking (also known as hookah, shisha, hubble bubble, and narghila) involves the passage of charcoal‐heated air through a perforated aluminum foil and across flavored tobacco (molasses) (Javed et al. 2017). This generates smoke which bubbles through water before being inhaled by the users (Fig. 1). Worldwide, tobacco smoking accounts for more than 6 million deaths per year and is expected to increase to 8 million deaths annually by 2030 (World Health Organization, 2011). Waterpipe smoking is commonly used in the Middle Eastern, Asian countries and is gaining popularity as a behavioral/recreational/social activity in western countries including the United States (Azab et al. 2010; Eissenberg et al. 2008; Maziak et al. 2015). Waterpipe tobacco users inhale smoke more intensively and deeply than cigarette smokers, suggesting that they inhale more toxic compounds. It is shown that waterpipe smoking generates similar concentration of particulate matter to cigarette smoking (Jacob et al. 2013; Jukema et al. 2014; Cobb et al. 2011; Eissenberg and Shihadeh 2009; Zhou et al. 2015). While there are substantial number of studies that relates to pulmonary toxicity of cigarette smoke (CS), but data on the lung health effects of waterpipe smoke (WPS) remains scarce (Knishkowy and Amitai 2005). Results from a meta‐analysis of six clinical studies showed a significant reduction in forced expiratory volume in 1 sec (FEV_1_) in subjects exposed to WPS compared with nonsmokers (Raad et al. 2011). Furthermore, acute exposure to WPS have been shown to cause a decrease in peak expiratory flow rate (PEFR) explaining a possible role of WPS in the progression of chronic obstructive pulmonary disease (COPD) (Hakim et al. 2011; Boskabady et al. 2012; She et al. 2014; Waked et al. 2011). Therefore, experimental studies involving animal model are much needed to study the underlying mechanisms of pulmonary toxicity caused by WPS.

The inbred strains of mice are genetically identical, thus different strains of mice may represent an ideal model to determine the susceptibility of acute toxicity on inflammatory and oxidative stress responses induced by WPS. A number of studies have used different mouse models showing varying degree of susceptibility to COPD/emphysema (Bartalesi et al. 2005; Cavarra et al. 2001; Guerassimov et al. 2004). It has been shown previously that oxidative stress and inflammatory responses induced by CS is strain‐dependent (Vlahos et al. 2006; Yao et al. 2008). However, the effect of WPS on lung oxidative and inflammatory responses in multiple strains of mice is not known. In this study, we used the two different mouse strains (C57BL/6J and BALB/cJ), and characterized the common and distinct features of the acute pulmonary toxicity on inflammatory and oxidative stress responses induced by WPS.

## Materials and Methods

### Ethics statement

We used a rigorous/robust and unbiased approach throughout the experimental plans for in vivo mouse model and during analyzing the results so as to ensure that our data are reproducible along with by full and detailed reporting of both methods and raw/analyzed data. Our results adhere to NIH standards of reproducibility and rigor.

### Animals

Adult C57BL/6J and BALB/cJ mice of both the sexes were purchased from the Jackson Laboratory (Bar Harbor, ME), (body weight 25–30 g; 14–16 weeks old) and were housed in inhalation core facility at the University of Rochester for 1 week acclimatization before waterpipe smoke (WPS) exposure. All animal procedures were approved by the University Committee on Animal Research of the University of the Rochester.

### Waterpipe smoke exposure

A waterpipe smoke whole body animal exposure system was designed as reported previously with modifications (Fig. 1) (Khabour et al. 2012). The exposure system contains two waterpipe smoking hookah setup connected to the FMI pump that draws the smoke and pumps into the exposure chamber. A computer controlled program was used to regulate the timed flow of WPS into the exposure chamber along with dilution air and exhausted through the HEPA‐HVAC system. The WPS passes through the water before it being drawn into the exposure chambers. Water was replaced daily before the exposure. The pump is setup to a defined puff topography using a computer controlled program (e.g., ~171 puffs/h, each puff is about 3–4 sec. long with 17 sec. puff interval) in accordance with the Beirut method with modifications (Khabour et al. 2012). During the exposure sessions, CO level in the chamber was monitored using data logger (El‐USB‐CO device) and total particulate matter (TPM) was monitored using gravimetric sampling. The flow rate of the pump which draws WPS was setup to 1.0 L/min with a constant flow of dilution air (filtered air) into the chamber (at the rate of 0.6 L/min). The exposure chambers were made of plexiglass, containing wired cages with cubical for individual mice. The average CO level in the chamber for 10 days WPS exposure was 630.96 ppm and average TPM in the chamber for WPS exposure measured from day 2–day 10 was 136 mg/m^3^.

Adult C57BL/6J and BALB/cJ mice were exposed to WPS for 10 consecutive days. Following 1 week of acclimatization, mice were randomly divided into air (control) and WPS group. Mice were exposed to 30 min of WPS twice a day with 1 h interval for conditioning high levels of CO during the first 3 days of exposure. For the remaining 7 days of WPS exposure, mice were exposed to 1 h WPS twice a day with 1 h interval. On an average 2–3 hookah charcoals were used for 1 h exposure duration/hookah setup. Approximately 12–13 gm of hookah tobacco molasses was used for each 1 h exposure in two separate hookah setup (Flavors used: Mixed fruit, Plum, Pipe, Cocktail and Nectarine). Mice were euthanized 24 h after the last WPS exposure. Blood COHb and CO levels were measured immediately after exposures. Control, age‐matched C57BL/6J and BALB/cJ mice (*n* = 8–12/strain) were exposed to filtered air, in an identical chamber according to the same protocol described for WPS exposure.

### I‐STAT System test cartridge for measuring blood gas analysis

Measurements for blood gas such as blood glucose, hematocrit, and hemoglobin were done using whole blood samples collected by submandibular venipuncture from air and WPS exposed mice immediately after the last day of exposure (Day 10) (Table 1). A hand held portable automatic i‐STAT test cartridge system (i‐STAT CG8+ cartridge Cat# 03P88‐25; Vet scan i‐STAT 1 analyzer; Abaxis Global Diagnostics) was used to perform blood gas analysis to measure, pH, pCO_2,_ PO2, HCO_3,_ TCO_2,_ sO2, Glu, Hct, and Hb.

**Table 1:** Blood gas analysis and hematology test results using venous blood samples by iSTAT system in C57BL/6J and BALB/cJ mice after 10 days of WPS exposure

Strains	pH	pCO_2_(mmHg)	PO_2_(mmHg)	HCO_3_(mmol/L)	TCO_2_(mmol/L)	sO2 (%)	Glu (mg/dl)	Hct (%PCV)	Hb (g/dl)
C57BL/6J									
AIR	7.3 ± 0.03	47.2 ± 3.58	34.5 ± 2.72	23.2 ± 0.81	24.5 ± 0.86	59.5 ± 3.79	191.7 ± 11.89	46 ± 0.57	15.6 ± 0.20
WPS	7.3 ± 0.01	43.4 ± 1.52	18.2 ± 1.37**	22.7 ± 0.78	23.7 ± 0.85	24.5 ± 2.95***	284.5 ± 20.74***	52.5 ± 0.64***	17.8 ± 0.23***
BALB/cJ									
AIR	7.3 ± 0.01	46.4 ± 3.35	32.6 ± 2.40	23.1 ± 0.86	24.3 ± 0.88	55.6 ± 4.33	148.3 ± 11.56	43.6 ± 0.33	14.8 ± 0.13
WPS	7.2 ± 0.03	42.5 ± 1.15	25 ± 1.73	20.4 ± 1.72	21.6 ± 1.85	38.6 ± 4.17#	136.3 ± 13.32	50.6 ± 0.88##	17.2 ± 0.29##

Data are expressed as mean ± SEM.

**P <* *0.001; ***P <* *0.0001 versus air exposed C57BL/6J.

^#^ *P *<* *0.05; ^##^ *P *<* *0.001 versus air exposed BALB/cJ.

### Bronchoalveolar lavage fluid (BALF)

After 24 h last exposure to 10 days of WPS or air exposures, mice were injected with pentobarbiturate (100 mg/kg body weight ip; Abbott Laboratories) and euthanized. The heart and lungs were removed en bloc, the trachea was cannulated and the lungs were lavaged three times with 0.6 mL of 0.9% NaCl. The recovered fluid aliquots were pooled. The lavaged fluid were centrifuged (1000*g* x 10 min, 4°C) and the cell free supernatant was stored at −80°C until further analysis. The BAL fluid was suspended in 1 mL of 0.9% NaCl and total cells counts/mL were determined by AO/PI staining using a cellometer. Differential cell counts (minimum of 500 cells per slide) were performed on cytospin‐prepared slides (Thermo Shandon, Pittsburgh, PA) stained with Diff‐Quik (Dade Behring, Pittsburg, PA).

### Plasma cotinine assay

Plasma cotinine in WPS exposed mice was measured to ensure that exposure to waterpipe smoking is equivalent. Blood collected from mice sacrificed immediately after 10 days of exposure was used to measure cotinine levels. Plasma cotinine assay was done by commercially available mouse/rat cotinine ELISA Kit as per the manufacturer's instructions (Calbiotech, Inc, Cordell Ct; El Cajon CA).

### Measurement of proinflammatory cytokines in the Bronchoalveolar lavage fluid (BALF)

The concentrations of 23 proinflammatory mediators from air/ WPS exposed mice were analyzed in the BAL fluid using the Bio‐Plex Pro mouse cytokine 23‐plex Immunoassay kit (Bio‐Rad Laboratory, Hercules, CA) according to the preoptimized protocol based on the methodology provided by the manufacturer.

### Determination of MPO levels in BAL fluid

Myeloperoxidase (MPO) in BAL fluid was measured by ELISA according to manufacturer's instructions (Cell Biolabs, Inc., San Diego, CA). MPO activity was expressed as mU/mL.

### Measurement of levels of reduced glutathione and oxidized glutathione in lung tissue

Total and oxidized (disulfide) glutathione levels were measured in mouse lung as described previously (Rahman et al. 2006a). Lung homogenates were prepared using 0.1 mol/L potassium phosphate (KPE) (pH 7.5) buffer with 0.5% sulfosalicylic acid. The concentration of protein in lung homogenate was measured by BCA kit and the levels were normalized and expressed as nmol/L of total glutathione/GSSG per mg protein.

### Measurement of lipid peroxidation products: 15‐isoprostane, 4‐hydroxy‐2‐nonenal and malondialdehyde

The concentration of 15‐isoprostane F_2t_ in the plasma was determined using a competitive ELISA kit according to the manufacturer's instructions (Oxford Biomedical Research, MI). The concentration of 15‐isoprostane F_2t_ in each sample was expressed as ng/mL. Measurement of the lipid peroxidation products 4‐hydroxy‐2‐nonenal (4‐HNE), and malondialdehyde (MDA) in lung homogenates was determined using HNE/ MDA adduct competitive ELISA kits as per the instructions from the manufacturer (Cell Biolabs, Inc. San Diego, CA). The values were expressed in samples as *μ*mol 4‐HNE/MDA/mg protein.

### Measurement of protein concentration

Protein level was measured with a Bicinchoninic acid (BCA) kit (Pierce, Rockford, IL).

### Statistical analysis

The Statistical analysis was performed using GraphPad Prism Software version 7.0. Statistical analysis of significance was calculated using two‐way ANOVA followed by Tukey's post hoc test for multiple comparisons. The results are shown as mean ± SEM unless otherwise indicated. *P *<* *0.05 was considered as statistically significant.

## Results

### Acute exposure of WPS did not affect the body weight

To determine whether 10 days acute WPS exposure influences any change in body weight, we assessed the body weight before and after exposures. In both the mouse strains (C57BL/6J & BALB/cJ), there was no significant difference in body weight measured before and after 10 days air or WPS exposure.

### Measurement of plasma cotinine

Blood collected from mice sacrificed immediately after 10 days of exposure was used to measure cotinine levels. Plasma cotinine levels in WPS exposed mice reached an average of 23.15 ± 5.89 ng/mL in C57BL/6J and 8.25 ± 3.56 ng/mL in BALB/cJ mice respectively (*P *<* *0.05). However, the plasma from mice sacrificed 24 h after last exposure did not show detectable cotinine levels in both the strains (data not shown).

### WPS exposure affected blood gas analysis

The effect of acute 10 days WPS exposure on blood gas was performed from venous blood collected by submandibular venipuncture using a i‐STAT system test cartridge (Table 1). Acute exposure to WPS induced a significant reduction (*P *<* *0.001) in PO_2_ in C57BL/6J mice compared to air exposed mice. Similarly, PO_2_ was decreased but not statistically significant (*P *=* *0.008) in BALB/cJ mice. The levels of PO_2_ in air and WPS exposed mice were lower than the arterial reference range (80–105 mmHg). This could be as a result of venous blood collected from mice immediately after day 10 of exposure to measure all the blood gas analysis instead of arterial blood. Oxygen saturation (sO_2_) decreased significantly in both the mouse strains. Following acute exposure to WPS glucose levels were increased significantly in C57BL/6J, but did not change in BALB/cJ mice. The hematocrit levels increased significantly in both the mouse strains compared with respective controls (Table 1).

### Inflammatory cell influx into the lungs of different strains of mice by WPS exposure

**Figure 2:**
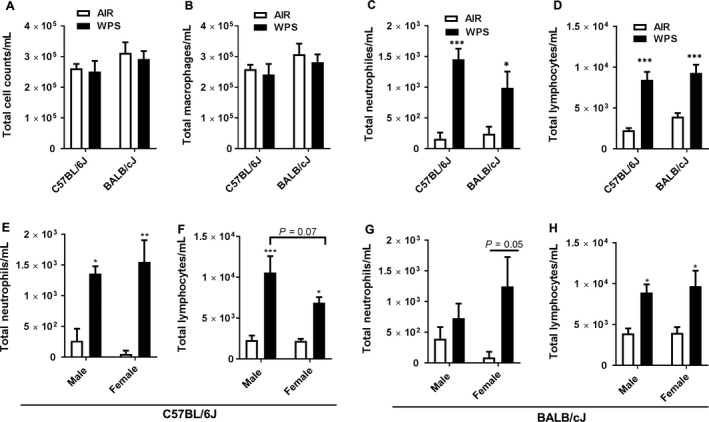
Acute WPS exposure induces pulmonary inflammation characterized by increased neutrophils and T‐lymphocytes in the BAL fluid. The inflammatory influx of (A) total cell counts, (B) macrophages, (C, E, and G) neutrophils, and (D, F, and H) lymphocytes into the BAL fluid of different strains of mice in response to 10 days WPS exposure. Total cells counts/mL were determined in BAL fluid by AO/PI staining using a cellometer. A total of 500 cells were counted to determine the total number of macrophages, neutrophils and lymphocytes on slides stained with Diff‐Quik. Data are represented as means ± SEM (*n* = 8 per group) and significance determined using two‐way ANOVA. *P < 0.05, **P < 0.01, ***P < 0.001 versus air.

We determined the inflammatory response in the BAL fluid of C57BL/6J and BALB/cJ mice after acute WPS exposure for 10 days. Total cell counts, including neutrophils, macrophages and lymphocyte counts were assessed by Diff Quik (Fig. 2). Acute WPS exposure significantly increased neutrophils and lymphocytes in the BAL fluid of both the strains (Fig. 2C–D). Of the two strains, WPS exposed C57BL/6J mice showed a modest inflammatory cellular influx with significant increase in neutrophils and lymphocytes in the BAL fluid compared to air exposed mice. Proportionally, C57BL/6J mice showed an increase in neutrophil counts in the BAL fluid compared to BALB/cJ mice.

Acute WPS exposure increased the total cell counts in BAL fluid of males, but not in females of both the mice strains (data not shown). Macrophage counts were decreased by WPS exposure in males and females of both C57BL/6J and BALB/cJ mice (data not shown). Sex‐based analysis clearly showed increased neutrophils in females of both the mice strains compared to males. Males and females from C57BL/6J mice showed increased neutrophil counts compared to males and females of BALB/cJ mice (Fig. 2E and G). Similarly, WPS exposed males and females from both the strains showed significant increase in lymphocyte counts (Fig. 2F and G).

### Inflammatory mediators in lungs of different strains of mice in response to acute WPS exposure

**Figure 3:**
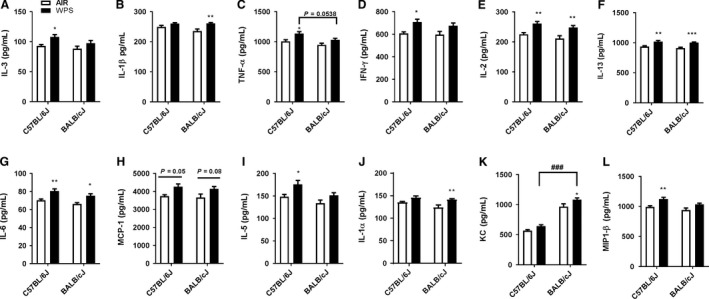
Acute WPS exposure increased proinflammatory cytokines release in the BAL fluid. C57BL/6J and BALB/cJ mice were exposed to air or WPS for 10 days and euthanized 24 h post last exposure. (A–L) Proinflammatory mediators from air or WPS exposed mice were measured in the BAL fluid by Luminex multiplex assay (Bio‐Rad). Data are shown as mean ± SEM (*n* = 8 per group) and significance determined using two‐way ANOVA. *P < 0.05, **P < 0.01, ***P < 0.001, versus air. ^# #^ *P < *0.01; ^# # #^ *P < *0.001 versus WPS.WPS, Waterpipe Smoke

Acute WPS induced proinflammatory cytokines were measured in different strains of mice as a measure of inflammatory response in the lungs. Proinflammatory mediators from air and WPS exposed mice were measured in the BAL fluid by Luminex multiplex assay (Fig. 3). The levels of IL‐3, tumor necrosis factor‐ alpha (TNF*α*) interferon gamma (IFN*γ*), IL‐5, macrophage inflammatory protein‐1*β* (MIP‐1*β*) and IL12p70 (data not shown) were significantly increased in WPS exposed C57BL/6J, but not in BALB/cJ mice (Fig.3A, C, D, I, and L). On the contrary, IL‐1*β*, IL‐1α, and keratinocyte chemoattractant (KC) were significantly (*P *<* *0.01) increased in response to WPS exposure in BALB/cJ, but not in C57BL/6J mice (Fig. 3B, J and K). However, there was no change in IL‐4, monocyte chemoattractant protein ‐1 (MCP‐1), IL‐10, granulocyte macrophage colony stimulating factor (GM‐CSF), macrophage inflammatory Protein ‐1*α* (MIP‐1α), and IL12p40 levels in both the mouse strains (data not shown). Remarkably, acute WPS exposure significantly increased levels of IL‐2, IL‐13, and IL‐6 in both the mouse strains suggesting these proinflammatory cytokines may play a critical role during WPS‐induced oxidative stress in the lungs (Fig. 3E–G). These results are in agreement with the inflammatory cell influx observed in the lungs of different mice strains following acute WPS exposure. Our findings suggest that WPS exposed C57BL/6J mice is more susceptible when compared to BALB/cJ mice based on release of proinflammatory cytokines.

**Figure 4:**
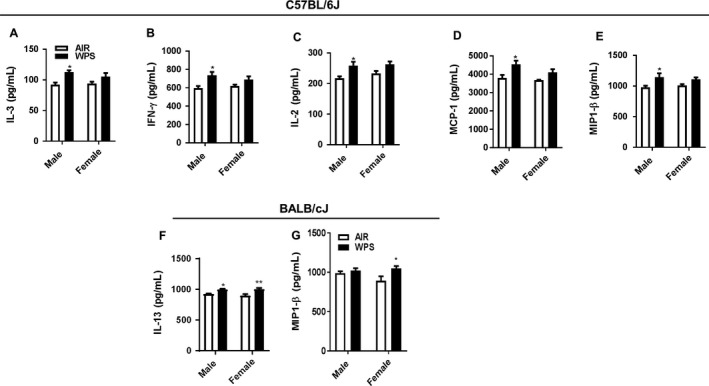
Effect of acute WPS exposure on proinflammatory mediators in male and female C57BL/6J and Balb/cJ mice. Male and female mice of (A–E) C57BL/6J and (F–G) BALB/cJ strains were exposed to air or WPS for 10 days and euthanized 24 h post last exposure. Proinflammatory mediators from air or WPS exposed mice were measured in the BAL fluid by Luminex multiplex assay (Bio‐Rad). Data are shown as mean ± SEM. (*n* = 4 per group) and significance determined using two way ANOVA. Significant statistical differences between the groups are indicated by symbols above the bars as: *Sex difference within the strain.*P < 0.05, **P < 0.01 versus air. WPS, Waterpipe Smoke

WPS exposure induced significant upregulation of IL‐3, IFN*γ*, IL‐2, MCP‐1 and MIP‐1*β* in males of C57BL/6J mice compared to respective air exposed mice (Fig. 4A–E). Females from C57BL/6J mice did not show significant changes in cytokines compared to respective air exposed mice. Thus, our data suggest that males are more susceptible to proinflammatory response compared to females in C57BL/6J mice. In BALB/cJ, only IL‐13 and MIP‐1*β* cytokines showed significant changes with respect to sex differences (males vs. females) (Fig. 4F–G). Interestingly, BALB/cJ mice exposed to WPS significantly increased IL‐13 release in both males and females, but MIP‐1*β* was significantly increased in only females as compared to respective air exposed mice. Others cytokines did not exhibit any significant changes with respect to sex in both the strains (data not shown).

### Levels of MPO in BAL Fluid

**Figure 5:**
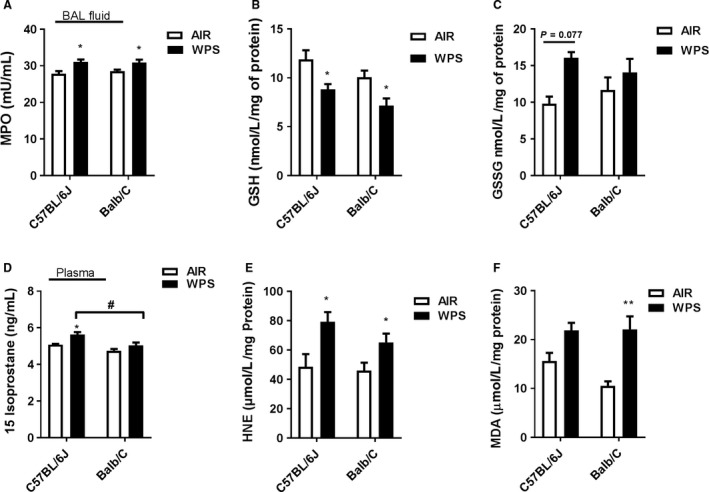
Acute WPS exposure alters oxidative stress markers in mouse Lung. C57BL/6J and BALB/cJ mice were exposed to air or WPS for 10 days and euthanized 24 h. post‐last exposure. (A) MPO in BAL fluid, (B) Reduced glutathione and (C) Oxidized Glutathione in lung homogenate, (D) 15‐isoprostane levels in plasma, (E) : 4‐Hydroxy‐2‐nonenal and (F) Malondialdehyde in lung homogenate were measured by ELISA according to manufacturer's instructions. Data are shown as mean ± SEM (*n* = 8–12 per group) and significance determined using two‐way ANOVA. *P < 0.05 versus air. #*P < *0.05 versus WPS.WPS, Waterpipe Smoke.

Myeloperoxidase is released by activated neutrophils, and the activity of MPO is used to estimate the level of neutrophilic inflammation in the tissues (Faith et al. 2008). WPS exposure significantly increased MPO levels measured in BAL fluid of both the mouse strains (Fig. 5A). Acute WPS exposure caused a moderate increase in MPO levels which was not statistically significant in both males and females of both the strains (data not shown).

### Levels of GSH and GSSG in lung tissue in response to acute WPS exposure

We assessed the effect of acute WPS exposure on intracellular reduced glutathione (GSH) and oxidized glutathione (GSSG) levels in both the strains of mice. Acute WPS exposure for 10 days resulted in significant decrease in GSH levels in both C57BL/6J and BALB/cJ mice (Fig. 5B). WPS exposure increased the GSSG levels in both mice strains though the difference was not statistically significant compared to air exposed control (Fig. 5C).

Similarly, total GSH level was increased in males and decreased in females in C57BL/6J and total GSSG level was increased in both the sexes (data not shown). We did not observe any significant difference with respect to sex in both the mouse strains. These results suggest WPS caused an imbalance in glutathione homeostasis as a result of altered GSH/GSSG levels in the lungs.

### Levels of lipid peroxidation products, 15‐isoprostane, 4‐HNE and MDA in response to acute WPS exposure

Plasma levels of 15‐isoprostane was increased in response to acute WPS exposure in both the mice strains compared to respective air control (Fig. 5D). However, significant difference was observed only in C57BL/6J, but not in BALB/cJ mice (Fig. 5D). Furthermore, we determined WPS induced lipid peroxidation products (4‐HNE and MDA) in different mice strains by ELISA. Our data showed that acute WPS exposure significantly increased the 4‐HNE levels in both the strains of mice compared to air control (Fig. 5E). Acute WPS exposure augments MDA levels in both the mice strains, however MDA level was significantly increased only in BALB/cJ mice (Fig. 5F). These finding demonstrates that WPS exposure elevates 4‐HNE and MDA levels in the lung of both the strains (Fig 5E and F).

Acute WPS exposure showed a moderate increase in 15‐isoprostane which was not statistically significant among males and females of both the mice strains (data not shown). The levels of 4‐HNE was increased in males and decreased in females of both the mice strains following WPS exposure compared to respective air controls. Similarly, MDA level was increased in both males and females of C57BL/6J and BALB/cJ strains compared to air control (data not shown). Interestingly, a significant increase in the levels of MDA in females of both the strains compared to males was observed. This suggests that the sex difference could contribute to overall degree of lipid peroxidation products released during WPS‐induced oxidative stress.

## Discussion

Previous studies using different mouse strains have demonstrated that oxidative stress and inflammatory response induced by cigarette smoke results in varying degree of susceptibility to COPD/emphysema (Bartalesi et al. 2005; Cavarra et al. 2001; Guerassimov et al. 2004; Vlahos et al. 2006; Yao et al. 2008). However, the effects of WPS exposure on pulmonary toxicity on oxidative stress and inflammatory responses particularly in different strains of mice are not studied. We hypothesized that different mouse strains show varying degree of susceptibility to WPS‐induced pulmonary toxicity. We studied the molecular mechanism of susceptibility to acute pulmonary toxicity induced by WPS using C57BL/6J and BALB/cJ mice. To the best of our knowledge, this study is first to report strain‐ and sex‐related differences with respect to pulmonary toxicity mediated by oxidative stress and inflammatory response following acute WPS exposure.

Acute WPS exposure resulted in an increased influx of neutrophils and lymphocytes in the BAL fluid of both the mice strains, although the degree of changes (proportion and numbers of neutrophils) varied. The neutrophil counts were significantly augmented in the BAL fluid of C57BL/6J compared to BALB/cJ mice. In general, C57BL/6J mice showed a modest increased in inflammatory response to acute WPS. The degree of inflammation due to WPS exposure observed in our study between the two strains was comparable to prior reports from acute CS exposure models. For example, Yao et al. (2008) reported that C57BL/6J mice were more sensitive to acute CS compared to A/J and 129/SvJ mice, whereas differential susceptibility was noticed by chronic CS exposures (Guerassimov et al. 2004; Rahman et al. 2017). The reason for this difference can be attributed to the fact we used WPS exposure in our model, and there may be some underlying inherent mechanistic differences between these two models with respect to degree of susceptibility in different strains.

In our study, 10 days acute exposure to WPS resulted in different quantitative release of proinflammatory mediators in both the mouse strains. C57BL/6J mice showed significant increase in several different T_H_1 and T_H_2 proinflammatory cytokines compared to BALB/cJ mice. The level of proinflammatory cytokines IL‐2, IL‐13, and IL‐6 were significantly increased in both the strains, suggesting that these cytokines can be considered as inflammatory biomarkers in BAL fluid of WPS exposed mice. CS exposure causes lung inflammation due to T_H_1 response in C57BL/6J and T_H_2 response in BALB/cJ mice (Churg et al. 2002). Our results are consistent with previous studies by Yao et al. (2008) and Vecchio et al. (2010) who also observed CS induced T_H_1 and T_H_2 proinflammatory cytokines in C57BL/6J and BALB/cJ mice. We also found acute exposure to WPS leads to an increase in T_H_1 response (IFN*γ*, TNFα, and IL‐2) in C57BL/6J and T_H_2 response (IL‐6 and IL‐13) in BALB/cJ mice. Furthermore, WPS exposure enhances recruitment of inflammatory cells and pro‐inflammatory cytokine release in C57BL/6J compared to BALB/cJ mice.

Myeloperoxidase has been widely used as a biomarker of lung inflammation and oxidative stress and constitutes the most abundant protein in neutrophils (Rahman et al. 2017). The level of MPO was augmented significantly in both the strains. Previous studies have shown the elevated levels of MPO in smokers (Vecchio et al. 2010), and mice exposed to WPS (Nemmar et al. 2015; Khabour et al. 2012; Nemmar et al. 2012, 2013). WPS mediated increase in neutrophilic inflammation leads to a heightened MPO levels in the lungs. This can also induce proinflammatory mediators, such as TNF*α*, IL‐6, IL‐8 and reactive oxygen species (ROS), thereby enhancing inflammatory response in the lung (Hoenderdos and Condliffe 2013).

Glutathione (GSH) is a key component of intracellular and extracellular antioxidant defense mechanisms in the lung. Protective role of GSH has been implicated in oxidative stress‐induced inflammation and lung injury (Rahman and MacNee 1999). We studied the effect of acute WPS exposure on intracellular GSH and GSSG levels in lungs of two different strains of mice. We showed that acute exposure to WPS resulted in significant decrease in GSH levels and increase in GSSG levels which was not significant in both the mice strains. This is likely due to more transient antioxidant adaptive response to WPS‐induced oxidative stress in C57BL/6J and BALB/cJ mice. We found that C57BL/6J strain was more susceptible to acute WPS‐induced oxidative stress response among the two strains. Our results are consistent with earlier findings that WPS induces oxidative stress by generation of ROS and depletion in GSH levels and increase in GSSG levels in several organs (Nemmar et al. 2013; Ali et al. 2017; Alzoubi et al. 2015; Rababa'h et al. 2016). It could also be possible due to the formation of GSH conjugates or rapid consumption of GSH during the breakdown of free radicals induced by WPS, thereby altering GSH redox system (Rahman and MacNee 1999). Overall, our findings on antioxidant parameters suggest that acute WPS exposure induces pulmonary toxicity by altering glutathione homeostasis.

An imbalance between radical‐generating and radical‐scavenging systems leading to inflammatory response and tissue injury has been associated with smoking (Rahman et al. 2006b). Lipid peroxidation produces a wide variety of oxidation products. F_2_‐isoprostanes are considered as one of the most reliable biomarker to assess oxidative stress in vivo. We found a significant increase in plasma 15‐isoprostane levels following acute WPS exposure in both the mouse strains. Furthermore, the levels of lipid peroxidation products (8‐isoprostane and 4‐HNE) are increased in EBC and lungs of patients with COPD (Montuschi et al. 2000; Rahman et al. 1996, 2002). 4‐HNE and MDA are among the many different aldehydes which are formed as secondary end products (lipid peroxidation products). Our data show that acute WPS exposure induced oxidative stress leading to an increase in 4‐HNE and MDA levels in the lungs of C57BL/6J and BALB/cJ mice. This observation is consistent with our previous findings that the levels of 4‐HNE were augmented in airways and alveolar epithelium of smokers and patients with COPD (Rahman et al. 2002). The increased levels of 4‐HNE and MDA in our study can be explained by the depletion of antioxidants mediated by WPS exposure. Furthermore, oxidant/antioxidant imbalance leads to sequestration of neutrophils that may result in oxidants burden and free radicals induced injurious responses in the lungs.

Previous studies have reported female smokers are more susceptible to increased risk of small airways disease compared to male smokers with severe COPD (Martinez et al. 2007). Also, female smokers experience an accelerated decline in lung function compared to male smokers with similar smoking exposure in mild to moderate COPD (Prescott et al. 1997). This difference can be attributed to pathologic changes influenced by female sex hormones (Prescott et al. 1997). We also investigated the sex‐related functional consequences of the acute WPS induced pulmonary toxicity on inflammatory and oxidative stress responses in two different mouse strains. Neutrophilic inflammation was more predominantly observed in females compared to males in both the strains. Also, total GSH levels decreased and GSSG levels increased more in females than males in both the strains. We did not observe any significant differences in pro‐inflammatory mediators and other oxidative stress parameters studied between the sexes. Our findings corroborated with previous human (Martinez et al. 2007; Prescott et al. 1997) and animal studies (Elliott et al. 2003, 2004) based on CS/nicotine exposures. Overall, males and females exhibited differential sensitivity for acute WPS exposure in the C57BL/6J and BALB/cJ mouse strains, and females exhibited increased susceptibility to pulmonary toxicity compared to males in both the strains.

The limitation of this study includes the use of only two commonly used inbred mouse strains. It would be interesting to compare oxidative stress and inflammatory parameters across multiple inbred mouse strains in response to chronic WPS exposure. In addition, the difference in susceptibility observed among the two different mouse strains could also be partly due to the method of exposures (nose‐only vs. whole body), duration, flavored hookah tobacco molasses, WPS setup conditions, which may have influenced some of the findings. Furthermore, we had less number of males versus females (3–4 mice/group) in both the strains which could have affected the outcome of some parameters measured.

In summary, following acute WPS exposure induced oxidative stress in both the mouse strains, and show increased inflammatory cell influx, proinflammatory mediator release, and lipid peroxidation products in the lungs. However, the extent of susceptibility to oxidative stress and inflammatory responses in the lungs was augmented in C57BL/6J compared to BALB/cJ mice, and more in female versus male mice. Overall, the C57BL/6J strain was highly sensitive, and can be used effectively to assess WPS exposure induced inflammation and oxidative stress in the lungs. In conclusion, acute WPS exposure induces pulmonary toxicity differentially in mice with diverse genetic backgrounds. Further study exploring these strain‐ and sex‐based differences and the molecular signaling mechanisms involved in determining chronic WPS induced pulmonary toxicity are warranted.

## Conflict of Interest

None declared.
